# Hesitancy towards COVID-19 Vaccines: An Analytical Cross–Sectional Study

**DOI:** 10.3390/ijerph18105111

**Published:** 2021-05-12

**Authors:** Abdelkarim Aloweidi, Isam Bsisu, Aiman Suleiman, Sami Abu-Halaweh, Mahmoud Almustafa, Mohammad Aqel, Aous Amro, Neveen Radwan, Dima Assaf, Malak Ziyad Abdullah, Malak Albataineh, Aya Mahasneh, Ala’a Badaineh, Hala Obeidat

**Affiliations:** 1 Department of Anesthesia and Intensive Care, School of Medicine, The University of Jordan, Amman 11942, Jordan; akaloweidi@hotmail.com (A.A.); s.halaweh@ju.edu.jo (S.A.-H.); m.al-mustafa@ju.edu.jo (M.A.); abo.3agel@hotmail.com (M.A.); awss369@gmail.com (A.A.); neveenradw@gmail.com (N.R.); Dima.assaf94@hotmail.com (D.A.); Black-diamond-8-@hotmail.com (M.Z.A.); Malakbataineh93@gmail.com (M.A.); ayamahasneh77@yahoo.com (A.M.); 2 Anesthesia and Intensive Care Department, Beth Israel Deaconess Medical Center, Harvard Medical School, Boston, MA 02215, USA; Asuleima@bidmc.harvard.edu; 3 Department of Anesthesia and Intensive Care, Prince Hamza Hospital, Amman 11947, Jordan; alaabadaineh2@gmail.com; 4 Maternal and Child Health Nursing Department, School of Nursing, Mutah University, Karak 61710, Jordan; Obeidathala@yahoo.com

**Keywords:** COVID-19, vaccine, social media

## Abstract

Vaccination is the most promising strategy to counter the spread of Coronavirus Disease 2019 (COVID-19). Vaccine hesitancy is a serious global phenomenon, and therefore the aim of this cross-sectional study was to explore the effect of educational background, work field, and social media on attitudes towards vaccination in Jordan. We compared between medical personnel who were in direct contact with patients and non-medical individuals at Jordan University Hospital in terms of demographics, knowledge about COVID-19 vaccines, rumors received via social media, their trust in these vaccines, and the encouraging factors for vaccination. 646 individuals were enrolled in this study, of which 287 (44.4%) were from medical field, and 359 (55.6%) from non-medical field. 226 (35%) were planning to take the vaccine once available, with a positive response from 131 (45.6%) medical field workers, compared to 94 (26.2%) non-medical individuals (*p* < 0.001). The social media rumor that was believed the most was the unsafety of these vaccines (*n* = 283; 43.8%). Only 163 (56.8%) of medical persons did not believe any of the circulated rumors, compared to 126 (35.1%) of non-medical persons (*p* < 0.001). The effect of medical personnel advice (OR = 0.83; 95% CI = 0.70 to 0.98; *p* = 0.026) and social media (OR = 1.21; 95% CI = 1.04 to 1.41; *p* = 0.012) were significantly associated with the willingness to take COVID-19 vaccine once available. In conclusion, medical personnel and social media play a crucial role in increasing the society’s inclination towards vaccination by providing the community with updated evidence-based information about COVID-19 vaccines as an efficient medical countermeasure and by correcting the previously spread misinformation.

## 1. Introduction

Coronavirus Disease 2019 (COVID-19) has caused a healthcare crisis all over the world. Social distancing, travel restrictions and self-isolation strategies have led to heavy socio-economic burdens. On a national basis, schools and factories closed, while demand for medical supplies and food increased tremendously, prompting healthcare providers to approach the pandemic with revolutionary solutions [1]. Since the declaration of the pandemic, countries are racing to slow down the spread of the virus by employing test regulations, contact tracing, travel bans, and lockdowns [2]. Today, vaccination tops the list of strategies to counter COVID-19 spread, with many approved marketed vaccines available for use [3].

Vaccine hesitancy is defined as delay in accepting or refusal of getting vaccination despite its availability [4]. Although the relative safety of marketed vaccines has been established [5,6,7], there are many other factors that affect community response towards vaccination, these include personal background (medical versus non-medical), social and political projections along with conspiracy theories, and safety concerns due to wide-spread myths and false messages spread on social media [8,9,10]. In Jordan, presently one of the countries most affected by the pandemic [11], the government has established an online portal to facilitate registration for vaccination. Despite many governmental campaigns on vaccination importance, the turnout on the portal was weak, uncovering alarming levels of hesitancy.

Previous studies showed that vaccine hesitancy is a rising global phenomenon with multifactorial background [12,13]. Most common related factors reported were safety issues, religious beliefs and lack of scientific knowledge [14]. In this study, we aim to explore the factors affecting attitudes towards vaccines at a tertiary hospital in Jordan, a middle eastern country. The primary objective is to study the effect of educational background and work field on responses towards vaccination. The secondary objectives are studying the most encouraging and discouraging factors to take the vaccine and how significant is the role of social media in shaping the attitudes of people in the Jordanian community.

## 2. Materials and Methods

### 2.1. Study Design

This correlational cross-sectional study was conducted between 22 January 2021 and 28 February 2021 using an online structured self-administered questionnaire. We included medical field workers from Jordan University Hospital (JUH), which is a tertiary teaching hospital in Amman, the capital of the Hashemite Kingdom of Jordan. The included medical personnel were those in direct contact with patients at inpatient or outpatient departments, including those who has history of COVID-19 and those who does not. Out of 2823 current employees at JUH, 2055 were considered in direct contact with patients, including physicians, nursing, pharmacists, technicians, and transporters [15]. Considering an acceptance rate of 28.4% for vaccination in Jordan [13], 5% precision, 95% confidence interval [23.4–33.4], and a standard error of 0.0255, a sample size of 282 was required from current JUH employees [16]. We reached 350 medical personnel, as well as 500 non-medical adult subjects as a comparator group. The response rate was 82% (287 participants) among medical field workers, and 71.8% (359 participants) among comparator group. The included participants in both groups were subjects who have not taken the vaccine yet, and do not have any family member who has been administered the vaccine yet. It is noteworthy to mention that Jordanian COVID-19 vaccination program was arranged by registering through an online platform [17]. Since the mid of January 2021, vaccination was scheduled using this automated registry, taking into consideration the age of the citizens, their medical illnesses, and their field of work [18].

### 2.2. Questionnaire

The questionnaire was designed using Google Forms, which is an online survey creator tool developed by Google. The questionnaire was divided into four sections. The first section included an introduction page, in which we introduced the topic and explained the study objectives and the participants consented to fill out the questionnaire by proceeding to the next section. The second section was the demographic section, in which we inquired about general demographic data and previous history of COVID-19 among the participants and their families. The third section questioned their knowledge about COVID-19 vaccines, their concerns regarding these vaccines, and the rating of their trust in those vaccines’ efficacy and safety on a scale of 10, where 10 signifies full trust, while 1 means minimal trust. In addition, this section also investigated their willingness to take the vaccine and to encourage others to take the vaccine. Moreover, it investigated the rate of influenza vaccine administration in the last year, as well as whether the participants consider themselves antivaxxers, which is a term that reflects a movement calling against vaccination in general [19].

In the fourth section, we investigated the most common rumors received via social media regarding COVID-19 vaccines, along with the most common circulated information regarding potential side effects. After that, we inquired about the most encouraging factors for vaccination, then the extent of influence of social media, medical personnel, teachers, professors and employers on the decision to get a vaccine was rated on a scale of 10, where 10 implies very high influence, while 1 means minimal influence. For the questionnaire validation, it was reviewed by 10 consultant physicians, after which it was modified based on their review. The calculated Cronbach’s alpha value was 0.76, indicating adequate internal consistency [20].

### 2.3. Ethical Approval

The study was approved by the Institutional Review Board (IRB) of JUH (approval No. 10-2021-4241). An informed consent was obtained at the end of the introduction page of the online questionnaire for the approval of participants to proceed to the questionnaire. We did not include any personal information, and the anonymously collected data was used solely for statistical analysis. 

### 2.4. Statistical Analysis

We used the Statistical Package for the Social Sciences (SPSS) version 25.0 (Chicago, IL, USA) for statistical analysis. Descriptive statistics were applied, and data was presented as mean ± standard deviation for numeric variables, and number (percent) for categorical variables. We used Mann-Whitney U test for the comparison between medical field workers and non-medical individuals in age, income, the aforementioned scales that measured the trust in the safety and efficacy of these vaccines, and scales measuring the extent of influence of social media, medical personnel, teachers, professors and employers on the decision to get a vaccine. Chi-squared (χ2) test was used to compare categorical variables between the aforementioned two groups. Univariable binary logistic regression analysis was applied to predict factors associated with willingness to administer COVID-19 vaccine, and the odds ratio (OR) as well as the 95% confidence interval (95% CI) of OR were demonstrated. Subsequently, the variables that were significant in the univariable regression model were then included in a multivariable binary logistic regression analysis. A two-sided *p*-value <0.05 was used as the significance threshold in all aforementioned statistical tests.

## 3. Results

Overall, 646 individuals were enrolled in this study, of which 169 (26.2%) were males, and 477 (73.8%) were females. The mean age of the studied population was 28.2 ± 10.8 years. Of the studied subjects, 133 (20.6%) have previous history of COVID-19. The demographic data and previous history of COVID-19 are illustrated in Table 1.

The comparison between medical and non-medical work fields showed no difference between the two groups in being concerned regarding getting COVID-19 in the future (*p* = 0.616) (Table 2). 137 (47.7%) of medical field workers have previously read a scientific article about COVID-19 vaccines, while only 112 (31.2%) of non-medical personnel have read such a scientific article (*p* < 0.001). The belief that COVID-19 vaccines are safe was higher among medical field workers (*n* = 163; 56.8%; *p* < 0.001) compared to non-medical persons (*n* = 118; 32.9%). The belief that those vaccines are able to protect against COVID-19 followed the same trend (*p* < 0.001), with 149 (51.9%) of medical personnel believing in the vaccine protection against this disease, compared to only 123 (34.3%) among non-medical persons. Upon inquiring about the most trusted vaccine according to the latest available information, 153 (42.6%) of non-medical persons did not have enough information to choose between the available vaccines (*p* < 0.001). Remarkably, medical staff were more concerned regarding the availability of the vaccines (*p* = 0.002).

Overall, 226 (35%) of the studied population were planning to take the vaccine once available. Of those who has previous history of COVID-19, 38 (28.6%) are planning to take the vaccine once available, while 95 (71.4%) were not. In comparison, 188 (36.6%) of those who did not have COVID-19 before were willing to take the vaccines (*p* = 0.086). Reading a scientific article about the available vaccines showed a significant increase in the rate of willingness to take the vaccines (*p* < 0.001), with 130 (52.2%) willingness rate among those who read a scientific article, compared to only 96 (24.2%) willingness to vaccinate among those who did not. Even among medical personnel alone, 85 (62.0%) of those who read a scientific article were willing to be vaccinated, compared to 47 (31.3%) willingness rate among those who did not read such an article (*p* < 0.001). 

Of the 287 medical field workers, 131 (45.6%) were willing to take the vaccine, compared to 94 (26.2%) of non-medical workers (*p* < 0.001). Moreover, 131 (45.6%) of the medical personnel and 94 (26.2%) of non-medical workers were willing to encourage others to take the vaccine (*p* < 0.001). Interestingly, 47 (13.1%) of non-medical staff considered themselves antivaxxers (*p* < 0.001), and only 37 (10.3%) of them took influenza vaccine (*p* < 0.001) (Table 2).

The circulated information and rumors about COVID-19 vaccines on social media platforms were illustrated in Table 3. The rumor that was believed the most was that these vaccines are not safe, with a prevalence of 283 (43.8%). This rumor was mostly believed by non-medical persons (*n* = 191; 53.2%; *p* < 0.001). On the other hand, 163 (56.8%) of medical persons did not believe in any of these circulated rumors, compared to 126 (35.1%) of non-medical persons (*p* < 0.001).

Upon investigating the most encouraging factors for vaccination, we found that short scientific videos were the most attractive method, being chosen by 390 (60.4%) persons. Social media awareness campaigns (*p* = 0.002), doctors’ advices (*p* = 0.009), television and radio-based advertisements (*p* = 0.024), national medical studies to prove vaccines efficacy (*p* = 0.009), and short scientific videos (0.004) were all chosen significantly more by medical field workers as an encouraging factor for vaccination, as illustrated in Table 4. The rating of trust the safety of these vaccines was higher among medical staff (5.2 ± 2.6) compared to non-medical field workers (4.1 ± 2.7; *p* < 0.001). In addition, the rating of the trust in efficacy of these vaccines (5.6 ± 2.7; *p* < 0.001) and the rating of trust in efficacy against mutated SARS-CoV-2 (4.5 ± 2.5; *p* < 0.001) were also higher in medical group, compared with 4.1 ± 2.8 and 3.5 ± 2.5 respectively among non-medical personnel. The rating of the effect of teachers, professors and employers’ advices on the decision to get a vaccine was also higher among medical staff, with a rating of 6.3 ± 2.3, compared to 5.9 ± 2.7 among non-medical personnel (*p* = 0.032).

We applied multivariable regression analysis (Table 5), in which we found that following factors are independently associated with the willingness to take COVID-19 vaccine once available: being concerned regarding getting COVID-19 in the future (OR = 1.82; 95% CI = 1.10 to 3.02; *p* = 0.02), the rating trust the safety of these vaccines (OR = 2.1; 95% CI = 1.72 to 2.56; *p* < 0.001), the rating of the trust in efficacy of these vaccines (OR = 1.34; 95% CI = 1.10 to 1.64; *p* = 0.003), the rating of the effect of medical personnel advice on the decision to get a vaccine (OR = 0.83; 95% CI = 0.70 to 0.98; *p* = 0.026), and the rating of the extent of social media effect on the decision to get a vaccine (OR = 1.21; 95% CI = 1.04 to 1.41; *p* = 0.012).

## 4. Discussion

Vaccines, when available, will likely be our best tool to counter the COVID-19 pandemic. Herd-immunity is one of the earliest ideas to come across at the beginning of the pandemic, but many studies showed that immunity acquisition from previous infection with COVID-19 is transient, and relatively inferior to immunity acquired through vaccination [21,22,23]. In our study the results showed no significant difference in willingness to take the vaccine in patients who were previously infected with COVID-19 when compared to those who were not. 

Previous studies assessing attitudes towards vaccines revealed huge regional variability in perceiving safety and effectiveness of marketed vaccines [24]. Interestingly, higher-income countries were the least certain regarding vaccine safety. Significant variability was noted on agreeing to vaccine safety between Northern America (72%), western Europe (59%) and eastern Europe (from 32% in Ukraine to 77% in Slovakia). However, majority of people in lower-income countries agreed on vaccination safety, with the highest proportions in South Asia (95%) and in Eastern Africa (92%) [25]. The assessment of these patterns can be invaluable in countering vaccine hesitancy.

Upon comparing people in medical and non-medical working fields, no significant difference was found regarding concern of getting COVID-19. Ideally, all healthcare workers should be worried about getting the infection as COVID-19 has been associated with increased mortality in these people [26]. Although there are other studies that showed no difference in terms of worrying [27], a previous study held in Jordan on frontline doctors dealing with COVID-19 related their concerns to lack of proper protective equipment [28]. 

Information resources about COVID-19 vaccines are many, including scientific journals, internet pages (blogs, news, and social media), friends, traditional media (television and radio), lessons on COVID-19 (whether video lessons or conference meetings), medical staff, and family members [29]. Scientific articles are labelled as the best source of information for medical and non-medical workers. In this study, there was a significant difference in reaching scientific articles between medical (47.7%) and nonmedical personnel (31.2%). Since COVID-19 is a relatively new pandemic and evidences with high level of confidence are being published only recently, one can expect the reluctance in reaching scientific articles as definite source of truth at the early stages of the pandemic. Nevertheless, the low percentage of reaching scientific articles among healthcare workers is worrying, as depression, anxiety, and distress are more likely to occur among healthcare workers if they were not exposed to the rightful information on COVID-19 [30].

Regarding safety of the vaccine, the belief that the vaccine is safe was higher among medical personnel (56.8%). This can be partially attributed to higher reach of scientific articles among medical personnel compared to non-medical ones. As a percentage, 56.8% is a relatively low percentage compared to other studies [31], but it points to the huge role other sources of information may play in the Jordanian community. 45.6% of medical field workers are planning to take the vaccine when available, compared to 26.2% of non-medical workers. The reported percentages among medical personnel worldwide ranged from 27.7% to 63% [32,33]. For non-medical group, only 50% of people in USA reported willingness to take the vaccine once available, while in France the percentage reached 74% [34]. Both medical and non-medical people showed readiness to encourage other people to take the vaccine with similar percentages. On the other hand, 13.1% considered themselves antivaxxers. Objections to vaccination can turn to strong attitudes if they are based on a religious background, associated with infringement of personal liberty, or being backed by suspicion of scientific articles [35].

The biggest challenge facing the employment of a vaccination strategy in any community is rumors [36]. In this study, the rumor that was believed the most was that the vaccines are not safe. There are many elements standing behind this rumor, most notably is the fake news spread over social media, described by the WHO (World Health Organization) as ‘infodemic’—indicating that the fake news spreads faster than the virus itself! [37]. Rumors on social media intermingles disinformation and misinformation about the COVID-19 vaccine, and even though many social media companies are employing strategies to fight these rumors, the spread is inevitable [38,39]. 46% of United Kingdom population and 48% in the united states reported exposure to rumors about COVID-19 and vaccines [40,41]. One of the most reported rumors among antivaxxers is that the virus is either manmade or produced by powerful organizations, and many people reported to believe in that in the United Kingdom and United States [40,42,43].

Regarding vaccination campaigns, the study found that the most effective method to encourage people to take the vaccine is short scientific videos spread on social media. Although one can argue with the definition of ‘scientific videos’, many experts around the world provide scientific knowledge through these platforms. The problem from vaccination perspective is that many of the social media published materials are claimed to be scientific, and till date, no efficient governmental or service provider strategies were able to validate this behavior [44,45]. 

Additionally, among the factors independently associated with the willingness to take COVID-19 vaccine are the rating of medical personnel advice on the decision to get a vaccine (OR = 0.83) and the rating of the extent of social media effect on the decision to get a vaccine (OR = 1.21). On a study targeting US adults’ attitudes towards COVID-19 vaccine, factors that were independently associated with hesitancy included younger age, lower educational level, and not having received the influenza vaccine in the prior year [46]. Another study implicated that perceived severity and fear of COVID-19 was associated with vaccine acceptance, while negative attitude towards general vaccination was associated with low vaccine acceptance [47]. 

During the data collection period of this study, the number of daily new cases in Jordan rose from 730 cases on 22 January 2021 to 4594 cases on 28 February 2021, after which the number of new cases continued to rise till it reached a peak of 9535 new cases on March 17th [11]. These dynamic changes in the numbers of new cases and their burden on the healthcare system and the society may influence the concern of the included persons of contracting COVID-19 and may eventually impact the vaccination acceptance rate.

Based on our results, we recommend that legal action should be taken by the government and the public security directorate in order to stop the spread of social media rumors and fake news about COVID-19 vaccines. Moreover, we encourage adequate funding for national medical studies to prove the safety and efficacy of these vaccines. Social media awareness campaigns are also encouraged, as social media was shown to have significant influence on the willingness to vaccinate against COVID-19. 

The main limitation of this study is that it did not investigate the awareness among individuals who does not use social media. Even though that recent estimates showed that most Jordanian adults own their own mobile phones [48], Those who do not use social media and depend on other sources for information should be considered in future studies. Moreover, the sample included in this study is not representative of the whole Jordanian population, for which future studies in which the distribution of occupation, age and, gender of the included sample should be similar to that of the country’s population. Furthermore, this study was conducted on individuals who have not taken the vaccine yet, and does not have any family member who has been administered the vaccine yet. Although this was considered a strength of this study by studying the influence of social media alone, the increasing numbers of vaccinated citizens in Jordan necessitates the need for investigating the influence of the feedback of family members, peers, and colleagues on the willingness to vaccinate for COVID-19. 

## 5. Conclusions

In conclusion, COVID-19 vaccines are considered the most promising intervention to control the spread of this pandemic. Vaccine hesitancy is a global challenge, and one of the main factors associated with it is the spread of misinformation on social media. In this study, we demonstrated that 35% of the studied population were planning to take the vaccine once available. The trust of the population in the safety and efficacy of these vaccines were positively associated with the willingness to vaccinate. Moreover, medical personnel and social media play a crucial role in increasing the inclination of the society towards vaccination. This imposes a huge responsibility on those sectors in providing the community with updated and evidence-based information about COVID-19 vaccines from trusted sources, in order to correct the previously spread misinformation and raise the population awareness regarding the importance of these vaccines as an efficient medical countermeasure.

## Figures and Tables

**Table 1:** Demographic data of the studied population.

Characteristic		Field of Work		Total	*p*-Value
		Medical Field (*n* = 287)	Non-Medical Field (*n* = 359)		
Age (years)		26.8 ± 8.9	29.2 ± 12.1	28.2 ± 10.8	0.518
Gender	Male	97 (33.8)	72 (20.1)	169 (26.2)	<0.001
	Female	190 (66.2)	287 (79.9)	477 (73.8)	
Marital status	Single	220 (76.7)	233 (64.9)	453 (70.1)	0.015
	Married	61 (21.3)	114 (31.8)	175 (27.1)	
	Divorced	4 (1.4)	8 (2.2)	12 (1.9)	
	Widowed	2 (0.7)	4 (1.1)	6 (0.9)	
Educational level	Elementary school	3 (1.0)	17 (4.7)	20 (3.1)	<0.001
	High school	9 (3.1)	46 (12.8)	55 (8.5)	
	Diploma	32 (11.1)	23 (6.4)	55 (8.5)	
	Bachelor	204 (71.1)	225 (62.7)	429 (66.4)	
	Masters	26 (9.1)	43 (12.0)	69 (10.7)	
	Doctor of Philosophy (PhD)	13 (4.5)	5 (1.4)	18 (2.8)	
Income (Jordanian dinars)		1103.2 ± 3151.6	943.5 ± 2764.8	1012.5 ± 2934.0	0.005
Area of residence	Urban	239 (83.3)	287 (79.9)	526 (81.4)	0.279
	Rural	48 (16.7)	72 (20.1)	120 (18.6)	
Previous history of COVID–19		61 (21.3)	72 (20.1)	133 (20.6)	0.708
Previous COVID-19 infection among family/friends		199 (69.3)	250 (69.6)	449 (69.5)	0.934
Lost someone due to COVID–19		79 (27.5)	135 (37.6)	214 (33.1)	0.007

Values are represented as number (percent) and mean ± standard deviation.

**Table 2:** Special concerns and general beliefs regarding available COVID-19 vaccines.

Characteristic	Field of Work		Total	*p*-Value
	Medical Field (*n* = 287)	Non-Medical Field (*n* = 359)		
Concerned regarding getting COVID-19 in the future	147 (51.2)	191 (53.2)	338 (52.3)	0.616
Heard about COVID-19 vaccines	286 (99.7)	354 (98.6)	640 (99.1)	0.169
Read a scientific article about COVID-19 vaccines	137 (47.7)	112 (31.2)	249 (38.5)	<0.001
Believe that COVID-19 vaccines are safe	163 (56.8)	118 (32.9)	281 (43.5)	<0.001
Believe that COVID-19 vaccines are able to protect them	149 (51.9)	123 (34.3)	272 (42.1)	<0.001
Believe that these vaccines have been sufficiently investigated	72 (25.1)	85 (23.7)	157 (24.3)	0.678
**The Most Trusted Vaccine According to the Latest Available Information**				
Oxford/AstraZeneca COVID-19 vaccine	15 (5.2)	26 (7.2)	41 (6.3)	<0.001
Sinovac COVID-19 vaccine	23 (8.0)	34 (9.5)	57 (8.8)	
Sputnik V COVID-19 vaccine	7 (2.4)	16 (4.5)	23 (3.6)	
Moderna COVID-19 Vaccine	13 (4.5)	13 (3.6)	26 (4.0)	
Pfizer–BioNTech COVID-19 Vaccine	149 (51.9)	117 (32.6)	266 (41.2)	
I do not know/ I do not have enough information	80 (27.9)	153 (42.6)	233 (36.1)	
**Concerns You Regarding Those Vaccines**				
1. Availability	70 (24.4)	61 (17.0)	131 (20.3)	0.02
2. Safety of packaging	79 (27.5)	81 (22.6)	160 (24.8)	0.146
3. Medical follow–up post vaccination	143 (49.8)	194 (54.0)	337 (52.2)	0.287
4. Effectiveness against mutated SARS–CoV–2	169 (58.9)	214 (59.6)	383 (59.3)	0.852
Planning to take the vaccine once available	132 (46.0)	94 (26.2)	226 (35)	<0.001
Will encourage others to take the vaccine	131 (45.6)	94 (26.2)	225 (34.8)	<0.001
Took influenza vaccine in the last year	83 (28.9)	37 (10.3)	120 (18.6)	<0.001
Took all your scheduled vaccines when you were a child	282 (98.3)	346 (96.4)	628 (97.2)	0.149
Consider themselves as antivaxxers	12 (4.2)	47 (13.1)	59 (9.1)	<0.001

Values are represented as number (percent).

**Table 3:** Circulated information about COVID-19 vaccines on social media platforms.

Characteristic	Field of Work		Total	*p*-Value
	Medical Field (*n* = 287)	Non-Medical Field (*n* = 359)		
**Rumors They Received via Social Media**				
It is unsafe	231 (80.5)	273 (76)	504 (78.0)	0.175
Effect of the vaccines on genetic level	126 (43.9)	149 (41.5)	275 (42.6)	0.54
Causes chronic illnesses	74 (25.8)	87 (24.2)	161 (24.9)	0.651
May lead to infertility	84 (29.3)	99 (27.6)	183 (28.3)	0.635
Can affect their offspring	57 (19.9)	73 (20.3)	130 (20.1)	0.881
Toxic heavy metals and neurotoxic materials	65 (22.6)	68 (18.9)	133 (20.6)	0.247
It is a part of a secret research	114 (39.7)	124 (34.5)	238 (36.8)	0.175
None of the above	34 (11.8)	47 (13.1)	81 (12.5)	0.635
**The Rumors That They Believed in**				
It is unsafe	92 (32.1)	191 (53.2)	283 (43.8)	<0.001
Effect of the vaccines on genetic level	25 (8.7)	62 (17.3)	87 (13.5)	0.002
Causes chronic illnesses	22 (7.7)	38 (10.6)	60 (9.3)	0.204
May lead to infertility	15 (5.2)	28 (7.8)	43 (6.7)	0.192
Can affect their offspring	24 (8.4)	32 (8.9)	56 (8.7)	0.805
Toxic heavy metals and neurotoxic materials	15 (5.2)	32 (8.9)	47 (7.3)	0.073
It is a part of a secret research	37 (12.9)	64 (17.8)	101 (15.6)	0.086
None of the above	163 (56.8)	126 (35.1)	289 (44.7)	0.001
**Side Effects They Heard about**				
local pain and swelling at site of injection	181 (63.1)	188 (52.4)	369 (57.1)	0.006
Fever	210 (73.2)	196 (54.6)	406 (62.8)	<0.001
Headache	156 (54.4)	131 (36.5)	287 (44.4)	<0.001
Fatigue	204 (71.1)	217 (60.4)	421 (65.2)	0.005
nausea	89 (31.0)	87 (24.2)	176 (27.2)	0.055
pain in the joints	87 (30.3)	78 (21.7)	165 (25.5)	0.013
Lymphadenopathy	57 (19.9)	36 (10.0)	93 (14.4)	<0.001
Fascial nerve palsy	39 (13.6)	48 (13.4)	87 (13.5)	0.936
anaphylaxis	105 (36.6)	96 (26.7)	201 (31.1)	0.007

Values are represented as number (percent).

**Table 4:** Factors affecting the willingness to get vaccinated for COVID-19.

Factor	Field of Work		Total	*p*-Value
	Medical Field (*n* = 287)	Non-Medical Field (*n* = 359)		
**Most Encouraging Factors for Vaccination**				
Social media awareness campaigns	177 (61.7)	178 (49.6)	355 (55.0)	0.002
Mandatory in schools, universities and workplaces	73 (25.4)	86 (24.0)	159 (24.6)	0.664
Mandatory for travelling	95 (33.1)	102 (28.4)	197 (30.5)	0.198
Vaccination campaigns	55 (19.2)	58 (16.2)	113 (17.5)	0.317
Doctors’ advices	148 (51.6)	148 (41.2)	296 (45.8)	0.009
Television and radio–based advertisements	82 (28.6)	75 (20.9)	157 (24.3)	0.024
National medical studies to prove their efficacy	188 (65.5)	199 (55.4)	387 (59.9)	0.009
National medical studies to prove their safety	173 (60.3)	195 (54.3)	368 (57.0)	0.128
**Most Influencing Social Media Tools to Encourage Vaccination**				
Online awareness posters	87 (30.3)	100 (27.9)	187 (28.9)	0.494
Influencers sharing their pictures while taking the vaccine	109 (38.0)	137 (38.2)	246 (38.1)	0.962
Short scientific videos	191 (66.6)	199 (55.4)	390 (60.4)	0.004
Short comedy videos	52 (18.1)	68 (18.9)	120 (18.6)	0.789
Sharing written information and posts using unified hashtags	46 (16.0)	59 (16.4)	105 (16.3)	0.889
Sharing pictures using unified hashtags	39 (13.6)	49 (13.6)	88 (13.6)	0.982
Competitions to raise awareness	36 (12.5)	44 (12.3)	80 (12.4)	0.912
Sponsored advertisements by non–governmental organizations	51 (17.8)	64 (17.8)	115 (17.8)	0.985
Rate how much you trust the safety of these vaccines	5.2 ± 2.6	4.1 ± 2.7	4.6 ± 2.7	<0.001
Rate how much you trust the efficacy of these vaccines	5.6 ± 2.7	4.1 ± 2.8	4.8 ± 2.8	<0.001
Rate how much you trust the efficacy against mutated SARS–CoV–2	4.5 ± 2.5	3.5 ± 2.5	3.9 ± 2.5	<0.001
Rating of the extent of social media effect on the decision to get a vaccine	7.9 ± 2.4	7.6 ± 2.6	7.7 ± 2.5	0.135
Rating of the effect of medical personnel advice on the decision to get a vaccine	7.1 ± 2.2	6.8 ± 2.5	6.9 ± 2.4	0.331
Rating of the effect of teachers, professors and employers’ advices on the decision to get a vaccine	6.3 ± 2.3	5.9 ± 2.7	6.1 ± 2.5	0.032

Values are represented as number (percent) and mean ± standard deviation.

**Table 5:** Univariable and multivariable regression analysis of factors affecting the willingness to take COVID-19 vaccine once available.

Variable	Univariable			Multivariable		
	OR	95% CI	*p*-Value	OR	95% CI	*p*-Value
Age	1	0.99–1.02	0.904	-	-	-
**Gender**						
Male	2.67	1.86–3.83	<0.001	1.02	0.56–1.85	0.949
Female	0.375	0.26–0.54	<0.001	0.98	0.54–1.78	0.949
Marital Status **			0.019			0.175
Married	0.55	0.37–0.80	0.002	0.47	0.24–0.92	0.028
Divorced	0.53	0.14–1.96	0.338	0.97	0.13–7.36	0.975
Widowed	0.79	0.14–4.34	0.783	0.53	0.03–9.28	0.663
**Field of Work**						
Medical field	2.4	1.73–3.34	<0.001	1.35	0.81–2.26	0.251
Non–medical field	0.417	0.30–0.58	<0.001	0.74	0.44–1.24	0.251
**Area of Residence**						
Urban	1.15	0.75–1.75	0.527	-	-	-
Rural	0.527	0.57–1.33	0.527	-	-	-
Income	1	1.00–1.00	0.419	-	-	-
Previous history of COVID-19 infection	0.69	0.46–1.05	0.083	-	-	-
Previous COVID-19 infection among family/friends	1.14	0.80–1.62	0.483	-	-	-
Lost someone due to COVID–19	1.36	0.97–1.91	0.076	-	-	-
Concerned regarding getting COVID-19 in the future	1.54	1.11–2.14	0.009	1.82	1.10–3.02	0.02
Rate how much you trust the safety of these vaccines	2.61	2.24–3.03	<0.001	2.1	1.72–2.56	<0.001
Rate how much you trust the efficacy of these vaccines	2.1	1.87–2.36	<0.001	1.34	1.10–1.64	0.003
Rate how much you trust the efficacy against mutated SARS–CoV–2	1.91	1.73–2.12	<0.001	1.03	0.86–1.23	0.746
Rating of the effect of medical personnel advice on the decision to get a vaccine	1.23	1.14–1.33	<0.001	0.83	0.70–0.98	0.026
Rating of the effect of teachers, professors and employers’ advices on the decision to get a vaccine	1.31	1.22–1.41	<0.001	1.05	0.91–1.21	0.526
Rating of the extent of social media effect on the decision to get a vaccine	1.33	1.22–1.45	<0.001	1.21	1.04–1.41	0.012

OR: odds ratio; 95% CI: 95% confidence interval. **: We used the marital status “single” as reference standard for all comparisons.

## Data Availability

The data presented in this study are available on request from the corresponding author.

## References

[1] Nicola M., Alsafi Z., Sohrabi C., Kerwan A., Al-Jabir A., Iosifidis C., Agha M., Agha R. (2020). The socio-economic implications of the coronavirus pandemic (COVID-19): A review. Int. J. Surg..

[2] (2020). United Nations Development Program. COVID-19 Pandemic Humanity Needs Leadership and Solidarity to Defeat COVID-19 Egypt: United Nations Development Programme. https://www.eg.undp.org/content/egypt/en/home/coronavirus.html.

[3] Rawat K., Kumari P., Saha L. (2021). COVID-19 vaccine: A recent update in pipeline vaccines, their design and development strategies. Eur. J. Pharmacol..

[4] MacDonald N.E. (2015). Vaccine hesitancy: Definition, scope and determinants. Vaccine.

[5] Yuan P., Ai P., Liu Y., Ai Z., Wang Y., Cao W., Xia X., Zheng J.C. (2020). Safety, Tolerability, and Immunogenicity of COVID-19 Vaccines: A Systematic Review and Meta-Analysis. medRxiv.

[6] Baden L.R., El Sahly H.M., Essink B., Kotloff K., Frey S., Novak R., Diemert D., Spector S.A., Rouphael N., Creech B. (2021). Efficacy and Safety of the mRNA-1273 SARS-CoV-2 Vaccine. N. Engl. J. Med..

[7] Voysey M., Clemens S.A.C., Madhi S.A., Weckx L.Y., Folegatti P.M., Aley P.K., Angus B., Baillie V.L., Barnabas S.L., Bhorat Q.E. (2021). Safety and efficacy of the ChAdOx1 nCoV-19 vaccine (AZD1222) against SARS-CoV-2: An interim analysis of four randomised controlled trials in Brazil, South Africa, and the UK. Lancet.

[8] Al-Qerem W.A., Jarab A.S. (2021). COVID-19 Vaccination Acceptance and Its Associated Factors Among a Middle Eastern Population. Front. Public Health.

[9] Daley M.F., Narwaney K.J., Shoup J.A., Wagner N.M., Glanz J.M. (2018). Addressing Parents’ Vaccine Concerns: A Randomized Trial of a Social Media Intervention. Am. J. Prev. Med..

[10] Arede M., Bravo-Araya M., Bouchard É., Gill G.S., Plajer V., Shehraj A., Shuaib Y.A. (2018). Combating Vaccine Hesitancy: Teaching the Next Generation to Navigate Through the Post Truth Era. Front. Public Health.

[11] Worldometers. Coronavirus Cases in Jordan. Dadax Ltd.. https://www.worldometers.info/coronavirus/country/jordan/.

[12] Wagner A.L., Masters N.B., Domek G.J., Mathew J.L., Sun X., Asturias E.J., Ren J., Huang Z., Contreras-Roldan I.L., Gebremeskel B. (2019). Comparisons of Vaccine Hesitancy across Five Low- and Middle-Income Countries. Vaccines.

[13] Sallam M. (2021). COVID-19 Vaccine Hesitancy Worldwide: A Concise Systematic Review of Vaccine Acceptance Rates. Vaccines.

[14] Karafillakis E., Larson H.J. (2017). The benefit of the doubt or doubts over benefits? A systematic literature review of perceived risks of vaccines in European populations. Vaccine.

[15] Jordan University Hospital (2019). Jordan University Hospital Annual Report 2019. http://hospital.ju.edu.jo/AnnualReports/Forms/All_Reports.aspx.

[16] Australian Bureau of Statistics: Sample Size Calculator. https://www.abs.gov.au/websitedbs/d3310114.nsf/home/sample+size+calculator.

[17] Jordanian Ministry of Health (2021). COVID-19 Vaccination Platform: MOH. https://vaccine.jo/cvms/.

[18] Omari R. (2021). Jordan Begins COVID-19 Vaccination Drive as Physician, 87, Gets First Jab Jeddah, 21433, Saudi Arabia: Arab News. https://www.arabnews.com/node/1791926/middle-east.

[19] Boodoosingh R., Olayemi L.O., Sam F.A. (2020). COVID-19 vaccines: Getting Anti-vaxxers involved in the discussion. World Dev..

[20] Tsang S., Royse C.F., Terkawi A.S. (2017). Guidelines for developing, translating, and validating a questionnaire in perioperative and pain medicine. Saudi J. Anaesth..

[21] Long Q.X., Liu B.Z., Deng H.J., Wu G.C., Deng K., Chen Y.K., Liao P., Qiu J.-F., Lin Y., Cai X.-F. (2020). Antibody responses to SARS-CoV-2 in patients with COVID-19. Nat. Med..

[22] Xiao A.T., Gao C., Zhang S. (2020). Profile of specific antibodies to SARS-CoV-2: The first report. J. Infect..

[23] Du Z., Zhu F., Guo F., Yang B., Wang T. (2020). Detection of antibodies against SARS-CoV-2 in patients with COVID-19. J. Med. Virol..

[24] Larson H.J., De Figueiredo A., Xiahong Z., Schulz W.S., Verger P., Johnston I.G., Cook A.R., Jones N.S. (2016). The State of Vaccine Confidence 2016: Global Insights Through a 67-Country Survey. EBioMedicine.

[25] (2019). Wellcome Trust. Wellcome Global Monitor: How Does the World Feel about Science and Health. https://wellcome.org/sites/default/files/wellcome-global-monitor-2018.pdf.

[26] Iyengar K.P., Ish P., Upadhyaya G.K., Malhotra N., Vaishya R., Jain V.K. (2020). COVID-19 and mortality in doctors. Diabetes Metab. Syndr..

[27] Parikh P.A., Shah B.V., Phatak A.G., Vadnerkar A.C., Uttekar S., Thacker N., Nimbalkar S.M. (2020). COVID-19 Pandemic: Knowledge and Perceptions of the Public and Healthcare Professionals. Cureus.

[28] Suleiman A., Bsisu I., Guzu H., Santarisi A., Alsatari M., Abbad A., Jaber A., Harb T., Abuhejleh A., Nadi N. (2020). Preparedness of Frontline Doctors in Jordan Healthcare Facilities to COVID-19 Outbreak. Int. J. Environ. Res. Public Health.

[29] Wang P.-W., Lu W.-H., Ko N.-Y., Chen Y.-L., Li D.-J., Chang Y.-P., Yen C.-F. (2020). COVID-19-related information sources and the relationship with confidence in people coping with COVID-19: Facebook survey study in Taiwan. J. Med. Internet Res..

[30] Lai J., Ma S., Wang Y., Cai Z., Hu J., Wei N., Wu J., Du H., Chen T., Li R. (2020). Factors Associated with Mental Health Outcomes Among Health Care Workers Exposed to Coronavirus Disease 2019. JAMA Netw. Open.

[31] Roy B., Kumar V., Venkatesh A. (2020). Health Care Workers’ Reluctance to Take the Covid-19 Vaccine: A Consumer-Marketing Approach to Identifying and Overcoming Hesitancy. NEJM Catal. Innov. Care Deliv..

[32] Nzaji M.K., Ngombe L.K., Mwamba G.N., Ndala D.B.B., Miema J.M., Lungoyo C.L., Mwimba B.L., Bene A.C.M., Musenga E.M. (2020). Acceptability of Vaccination Against COVID-19 Among Healthcare Workers in the Democratic Republic of the Congo. Pragmatic Obs. Res..

[33] Kuter B.J., Browne S., Momplaisir F.M., Feemster K.A., Shen A.K., Green-McKenzie J., Faig W., Offit P.A. (2021). Perspectives on the receipt of a COVID-19 vaccine: A survey of employees in two large hospitals in Philadelphia. Vaccine.

[34] Cornwall W. (2020). Just 50% of Americans Plan to Get a COVID-19 Vaccine. Here’s Kow to Win over the Rest. Science. https://www.sciencemag.org/news/2020/06/just-50-americans-plan-get-covid-19-vaccine-here-s-how-win-over-rest.

[35] Ashton J. (2021). COVID-19 and the anti-vaxxers. J. R. Soc. Med..

[36] Geoghegan S., O’Callaghan K.P., Offit P.A. (2020). Vaccine Safety: Myths and Misinformation. Front. Microbiol..

[37] United Nations (2020). UN Tackles ‘Infodemic’ of Misinformation and Cybercrime in COVID-19 Crisis. https://www.un.org/en/un-coronavirus-communications-team/un-tackling-%E2%80%98infodemic%E2%8099-misinformation-and-cybercrime-covid-19.

[38] Gellin B. (2020). Why vaccine rumours stick—And getting them unstuck. Lancet.

[39] van der Linden S., Roozenbeek J., Compton J. (2020). Inoculating against Fake News About COVID-19. Front. Psychol..

[40] Roozenbeek J., Schneider C.R., Dryhurst S., Kerr J., Freeman A.L.J., Recchia G., van der Bles A.M., van der Linden S. (2020). Susceptibility to misinformation about COVID-19 around the world. R. Soc. Open Sci..

[41] Mitchell A., Oliphant J.B. (2020). Americans Immersed in COVID-19 News; Most Think Media Are Doing Fairly Well Covering It: Pew Research Center. https://www.journalism.org/2020/03/18/americans-immersed-in-covid-19-news-most-think-media-are-doing-fairly-well-covering-it/.

[42] Freeman D., Waite F., Rosebrock L., Petit A., Causier C., East A., Jenner L., Teale A.-L., Carr L., Mulhall S. (2020). Coronavirus conspiracy beliefs, mistrust, and compliance with government guidelines in England. Psychol. Med..

[43] Uscinski J.E., Enders A.M., Klofstad C., Seelig M., Funchion J., Everett C., Wuchty S., Premaratne K., Murthi M. (2020). Why do people believe COVID-19 conspiracy theories?. Harv. Kennedy Sch. Misinf. Rev..

[44] Smith T.C., Reiss D.R. (2020). Digging the rabbit hole, COVID-19 edition: Anti-vaccine themes and the discourse around COVID-19. Microbes Infect..

[45] Scerri M., Grech V. (2020). WITHDRAWN: COVID-19, its novel vaccination and fake news—What a brew. Early Hum. Dev..

[46] Fisher K.A., Bloomstone S.J., Walder J., Crawford S., Fouayzi H., Mazor K.M. (2020). Attitudes Toward a Potential SARS-CoV-2 Vaccine: A Survey of U.S. Adults. Ann. Intern. Med..

[47] Qiao S., Tam C.C., Li X. (2020). Risk exposures, risk perceptions, negative attitudes toward general vaccination, and COVID-19 vaccine acceptance among college students in South Carolina. medRxiv.

[48] Al-Hadidi F., Bsisu I., AlRyalat S.A., Al-Zu’Bi B., Bsisu R., Hamdan M., Kanaan T., Yasin M., Samarah O. (2019). Association between mobile phone use and neck pain in university students: A cross-sectional study using numeric rating scale for evaluation of neck pain. PLoS ONE.

